# Next Generation Sequencing-Based Investigation of Potential Patient-to-Patient Hepatitis C Virus Transmission during Hemodialytic Treatment

**DOI:** 10.1371/journal.pone.0147566

**Published:** 2016-01-25

**Authors:** Qi Zhao, Yujie Wen, Yan Jiang, Chen Zhang, Yang Li, Guiyun Zhang, Lei Zhang, Maofeng Qiu

**Affiliations:** 1 National AIDS Reference Laboratory, National Center for AIDS/STD Control and Prevention, Chinese Center for Disease Control and Prevention, Beijing, China; 2 National Institute for Viral Disease Control and Prevention, Chinese Center for Disease Control and Prevention, Beijing, China; 3 Research Center for Public Health, School of Medicine, Tsinghua University, Beijing, China; 4 Melbourne Sexual Health Centre, Alfred Health, Melbourne, Victoria, Australia; 5 Central Clinical School, Faculty of Medicine, Nursing and Health Sciences, Monash University, Melbourne, Victoria, Australia; University of Cincinnati College of Medicine, UNITED STATES

## Abstract

We investigated potential patient-to-patient transmission of hepatitis C virus (HCV) in two hemodialysis centers in Beijing, China. Approximately 8.25% (32/388) hemodialysis patients were HCV antibody positive, and 4.90% (19/388) were HCV RNA-positive, which consisted of 2a genotype (1/19) and 1b genotypes (18/19). Using next generation sequencing (NGS) approach, MiSeq platform, we sequenced HCV, targeting hypervariable region 1 (263 base-pairs) of genotype 1b specimens and obtained 18 to 243 unique HCV variants. Analysis of phylogenetic tree, viral epidemiology signature pattern (VESP) and Shannon entropy indicated no obvious HCV similarity for most HCV infections but limited HCV variants from Patient 31 (P31) were closer with respect to evolutionary relationship with Patient 24 (P24). However, it was unlikely that HCV was transmitted directly from P24 to P31 in the hemodialysis center. Otherwise, their genetic distance (3.92%-8.92%), would have been much less. Moreover, P31 was infected less than two years before specimen collection, and other external high risk factors existed for these two patients. Thus, our data indicated no evidence of patient-to-patient transmission of HCV in the two hemodialysis centers, suggesting that current HCV infection control measures are effective.

## Introduction

An estimated 7.01% patients who receive hemodialysis are anti-HCV positive in China [1] and this prevalence rate- while lower than previous rates- exceeds that in the general population (0.43%, [2]). As reported previously, intra-facility HCV transmission has been increasingly recognized as a risk for hemodialysis patients, especially when HCV infections *via* blood transfusion or surgery have decreased dramatically due to rigorous routine screening policies [3, 4]. In 2011, a study to investigate HCV prevalence in 20 hemodialysis centers was launched in China [5] and 2120 patients were enrolled. Of these subjects 6.08% were anti-HCV positive and medical records confirmed that HCV seroconversion occurred while patients received long-term hemodialysis in two separate centers.

Because other external high risk factors existed for these subjects, such as blood transfusion and surgery, concluding that hemodialysis was the infection source was difficult. To investigate the potential for patient-to-patient transmission we studied patient data to identify potential risks during hemodialysis at both centers and to propose measurements to prevent new HCV infections.

Molecular evolutionary analyses have been used to assess genetic similarities and to investigate transmission of clinical pathogens [6–9]. However, tracing HCV infectious sources that are associated with hemodialysis is difficult because patients may be re-infected with HCV strains from different sources. Therefore, analysis based on limited sequences may be unsuitable for our studies. Detection of sufficient variants with molecular approaches such as post-cloning polymerase chain reaction (PCR) and end-point limiting-dilution (EPLD)-PCR are useful but time-consuming and labor-intensive [10, 11]. In comparison, next generation sequencing (NGS) approaches can identify HCV in populations with greater sequence depth accuracy [12, 13] and NGS techniques have a low cost per base and provide more detailed sequence information, making them more attractive than molecular analytical assays that require more sequencing depth.

Thus, we developed an NGS approach (MiSeq platform) for tracing HCV infection, and investigated potential HCV patient-to-patient infections at two hemodialysis centers in Beijing, China. Because other patients also attended the same centers regularly, we could compare subjects to determine whether HCV infections were due to patient-to-patient transmission within centers and reveal potential deficits in infection control measurements.

## Materials and Methods

All hemodialysis patients (N = 195 patients, Center A; 193 patients, Center B) were enrolled from July to November 2011. Serological testing indicated that 15 patients (7.69%) in Center A (P1-P15) and 17 patients (8.81%) in Center B (P16-P32) were anti-HCV positive. HCV RNA qualitative testing confirmed HCV RNA-positive rates for patients in Center A and Center B were of 4.10% (8/195) and 5.99% (11/193), respectively. All hemodialysis patients were anti-HIV negative and 3 patients (P19, P22, P31) were infected with HBV prior to hemodialysis. Given that no epidemiological relatedness was found between patients from the two centers, patients from one center was a control for phylogenetic analysis for patients from the other center. Information for 19 patients, including possible risk factors, medical records, and patient demographics were collected. Ethical approval to conduct this study was granted by the Institutional Review Board of the National Center of AIDS/STD Control and Prevention, Chinese Center for Disease Control and Prevention. Written informed consent was obtained from all participants for their clinical records to be used in this study.

To identify viral genotypes, the HCV NS5B region (H77 positions: 8276–8615) and core/E1 region (869–1292) were amplified with nested PCR and then sequenced directly using Sanger’s method. Primers and PCR conditions have been previously published [14, 15]. The MiSeq platform (Illumina, Inc.) was used to measure intra-personal HCV diversity of hypervariable region-1 (HVR-1:1321–1583). Targeted fragments were amplified with nested PCR and Q5 Hot Start High-Fidelity DNA Polymerases (New England Biolabs). Primers were optimized according to published methods [10] and specimen genotypes were as follows: external primers, H1-TGG CTT GGG ATA TGA TGA TGA ACT (1293–1316) and H2- GCA GTC CTG TTG ATG TGC CA (1598–1617); internal primers, H3-GGA TAT GAT GAT G CTG GT (1301–1320) and H4-ATG TGC CAR CTG CCR TTG GTG T (1584–1605).

PCR for the first round was: 30 s at 98°C; followed by 30 cycles for 10 s at 94°C, 30 s at 67°C, and 2 min at 72°C. The second round was identical to the first round, except the annealing temperature was reduced to 60°C. Paired-end sequencing of 250 base-pairs was performed on a MiSeq platform. Sequencing libraries were built using Nextera XT DNA Sample Preparation Kit (Illumina, Inc.). A data cleaning strategy was performed according to published methods [16]. We added another step to improve sequencing quality: sequences appearing less than 30 times were discarded. Nucleotide sequences were aligned using the HCV Align program of the HCV database (http://hcv.lanl.gov/content/sequence/VIRALIGN/viralign.html). MAFFT [17]. Forty representative sequences, with identical genotypes to specimens, were downloaded from the HCV database (http://www.hcv.lanl.gov/) and worked as external controls. Genetic distance and a neighbor-joining (NJ) tree were calculated with a Kimura 2-parameter model (provided by MEGA6.06). Statistical differences of inter-personal distances were tested with SPSS 19.0 software using a Mann-Whitney *U* test. P < 0.05 was viewed as significant. Viral epidemiology signature pattern (VESP) analysis and Shannon entropy-two analysis were calculated with online tools available at the HCV database.

## Results

All HCV RNA-positive specimens were amplified successfully using nested PCR and subsequently genotyped. Genotypes of HCV from P26 was 2a and the other 18 were 1b. The transmission possibility between P26 and other patients could therefore be excluded. As a result, only 18 specimens with HCV genotype 1b were subsequently analyzed by NGS. We summarized the primary characteristics of 19 patients who were HCV RNA-positive in Table 1. The average hemodialysis duration for these HCV patients was 15 years (ranging 3–25 years), and two patients underwent less than ten-year treatment. Three patients (P21, P22, and P30) had been infected with HCV prior to their first hemodialysis treatment. The other 16 patients were infected with HCV during hemodialysis, but their transmission modes remained unclear.

**Table 1:** Epidemiological and molecular information about HCV RNA-positive subjects.

Patient	Duration of hemodialysis (yr)	Initial time of hemodialysis	Current hemodialysis center^b^	Possible risk factors^c^				Confirmation of HCV infection	Genotype	No. of Unique variants	Intrapatient genetic distance (%)
				Blood transfusion (frequencies)	Reuse of hemodialysis machines	Hemodialysis in different centers	Operation history				
P2	13	1998.7	A (1998.7)	-	Y	-	-	2009.7	1b	115	2.88 (0.38–6.38)
P3	N/A^a^	N/A^a^	A (N/A^a^)	Y (N/A^a^)	Y	-	Y	2011.8	1b	145	1.00 (0.38–2.32)
P4	25	1987.1	A (1992.1)	Y (>10)	Y	Y	Y	2011.8	1b	87	1.53(0.38–3.53)
P7	16	1995.1	A (1995.1)	Y (1)	Y	-	Y	1998.1	1b	51	1.01 (0.38–2.33)
P8	14	1997.8	A (1997.8)	Y (1)	-	-	-	2010.1	1b	138	1.79 (0.38–5.57)
P11	17	1994.2	A (1994.2)	Y (3)	-	-	-	2008.1	1b	130	2.06 (0.38–6.36)
P13	12	2000.1	A (2000.1)	Y (1)	-	-	-	2008.1	1b	243	2.37 (0.38–6.87)
P15	19	1993.1	A (1993.1)	Y (>10)	Y	Y	Y	2009.5	1b	27	0.80 (0.38–1.53)
P16	20	1991.7	B (1999.9)	Y (1)	Y	Y	Y	1991.9	1b	109	3.06 (0.38–6.87)
P18	12	1999.4	B (2000.4)	-	Y	Y	Y	2002.4	1b	116	2.98 (0.38–6.01)
P19	12	1999.11	B (1999.11)	Y (2)	Y	-	Y	2000.5	1b	58	1.26 (0.38–2.70)
P21	14	1997.5	B (2003.5)	Y (3)	Y	Y	Y	1997.1	1b	95	2.70 (0.38–6.38)
P22	3	2008.8	B (2008.8)	Y (6)	-	Y	Y	2008.1	1b	38	1.64 (0.38–4.73)
P24	20	1992.1	B (2006.5)	Y (2)	-	Y	Y	2006.5	1b	92	1.66 (0.00–5.19)
P25	20	1991.4	B (2009.1)	Y (>10)	Y	Y	Y	1992.7	1b	35	1.07 (0.38–2.32)
P26	11	2000.5	B (2009.11)	Y (N/A^a^)	Y	Y	Y	2007.7	2a	Not detected	Not detected
P28	14	1997.3	B (1997.3)	-	Y	-	Y	2008.6	1b	18	0.80(0.38–1.53)
P30	8	2003.5	B (2008.5)	-	-	Y	Y	2003.1	1b	76	1.73 (0.38–5.10)
P31	13	1999.1	B (2003.5)	Y (>10)	Y	Y	Y	2011.2	1b	123	2.55 (0.38–8.98)

^a^ Duration of hemodialysis, initial hemodialysis date, the initial date of attendance to current center and renal transplantation date were unclear for this patient.

^b^ The initial date of attendance to current center were added in the parenthesis.

^c^ This study revealed four kinds of high risk factors. If one patient had one of the risk factors, then this factor was marked with “Y.” If not, then it was marked with “-”

**Fig 1:**
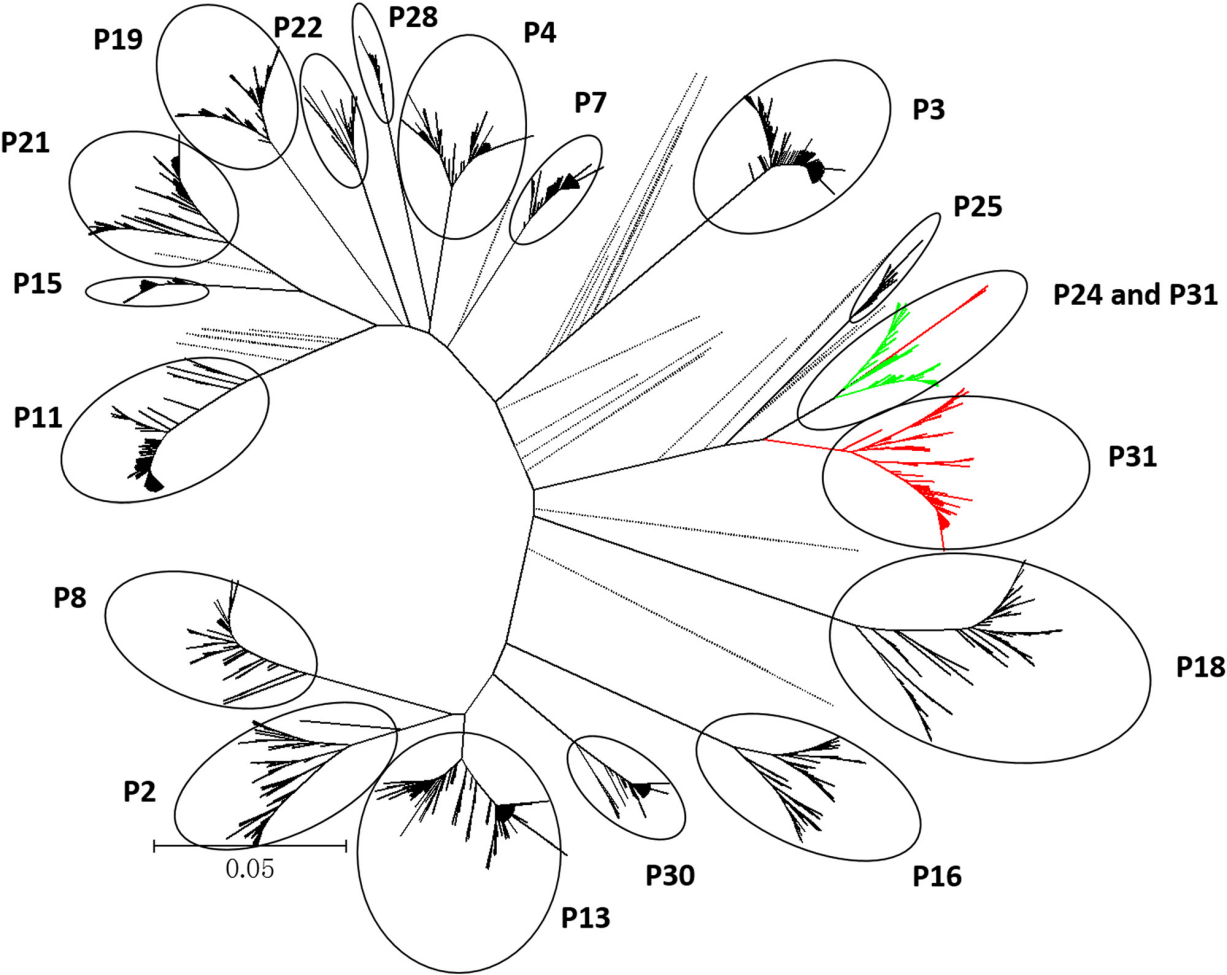
Phylogenetic tree analysis on the HCV sequences from 18 patients with HCV genotype 1b. The Neighbor-joining (NJ) tree was built based on the HCV HVR-1 region (H77 positions: 1321–1583) from 18 hemodialysis patients. A bootstrap test was performed (n = 1000) and statistic values > 70% were shown. Variants from each specimen were labeled with black dotted circles, whereas variants from related patients (P24, P31) were colored, respectively. Forty HCV representative sequences from the Los Alamos National Laboratory HCV database were shown in black discontinuous lines.

An average of 94 unique HCV variants (ranged 18–243) from each specimen was determined by NGS (GenBank accession numbers: KR909903-KR911598). The genetic distance between any two patients in Center A ranged from 7.53% (between P2 and P13) to 27.48% (between P3 and P8). The genetic distance between patients in Center B ranged from 6.51% (between P24 and P31) to 27.27% (between P16 and P18). The closest distance between any patients in Center A and another patient in Center B was 8.90%. The phylogenetic tree indicated a relatively close evolutionary relationship of two patients, P24 and P31, who received hemodialysis at Center B (Fig 1). Other specimens were in separate clusters, which were isolated from each other by HCV representative sequences respectively.

VESP analysis confirmed that 11 sequence sites on P24’s signature pattern were significantly different from that of the 6 variants of P31. However, there were 15 significantly different sites between the signature pattern of P24 and P31’s remaining variants. The number of different sites between P24 and any controls was from 40 to 57. Similarities could be observed between P31 and controls, which ranged from 41 to 57. Shannon Entropy-Two analysis of P24 and the 6 variants of P31 suggested that 7 out of 263 sequence sites have stronger variable characteristic, with 4 in P24 and 3 in P31. A less conserved trend (23/263) was observed between P24 and P31’s remaining variants, with 13 variable sites in P24 and 10 in P31.

## Discussion

We investigated HCV status and genotypes among patients receiving hemodialysis at two separate centers (A and B) from July to November 2011. Anti-HCV positive rates for patients in both centers were consistent with published data [1]. Phylogenetic tree analysis confirmed no obvious HCV similarity for most patients in the same center and they were divided into separate clusters by representative sequences. Thus patient-to-patient transmission within the two centers was not supported. Epidemiological surveys revealed at least two types of risk factors outside of the hemodialysis centers which may explain HCV infections: multiple blood transfusion and surgery. Phylogenetic tree analysis suggested that limited HCV variants from P31 occupied a close spatial position with P24. Shannon Entropy-Two analysis also showed that P24 exhibited a more conserved evolutionary pattern with those 6 variants of P31 than with the remaining variants. VESPA data indicated that the remaining P31 variants were closer epidemiologically with P24 than unrelated controls, supporting a relationship between HCV for P24 and P31. Medical records indicated that P31 was anti-HCV positive nine months prior to specimen collection. Considering the routine HCV screening program at the hemodialysis centers (every six months) and the window period for anti-HCV testing, P31 may be infected with HCV within two years prior to specimen collection. The average distance between P24 and P31, however, was larger than what (0.00%-1.48%) in other short—term infections [11, 18], which might indicate a long-term infection. Further, there was no recent cross-contamination of HCV between the two patients. Given other external high risk factors, HCV similarity between the two patients may be due to external infection and treatment at the same center could be likely coincidental. High-risk factors contributing to HCV infections in hemodialysis centers include the use of a single machine by multiple patients, inadequate machine and surrounding environment disinfection, and dialysis fluid contaminated by HCV [4, 19]. However, our investigation suggests that the current control measurements for HCV infection and routine screening programs are reliable.

We obtained more unique variants with an NGS approach compared with conventional methods [11, 20] and we had fewer technical difficulties for detecting low frequent sequences from P31. Thus, we could better identify real genetic relationship between two patients. NGS allows algorithm development, pipeline simplicity for data analysis, and labor- and time-saving procedures for sequencing. Our approach may facilitate HCV epidemiologic investigations and identification of infection risks during long-term hemodialysis.
